# Revised phylogeny and historical biogeography of the cosmopolitan aquatic plant genus *Typha* (Typhaceae)

**DOI:** 10.1038/s41598-018-27279-3

**Published:** 2018-06-11

**Authors:** Beibei Zhou, Tieyao Tu, Fanjiao Kong, Jun Wen, Xinwei Xu

**Affiliations:** 1 National Field Station of Freshwater Ecosystem of Liangzi Lake, College of Life Sciences, Wuhan University, Wuhan, 430072 PR China; 2 Key Laboratory of Plant Resources Conservation and Sustainable Utilization, South China Botanical Garden, Chinese Academy of Sciences, Guangzhou, 510650 PR China; 3 Department of Botany, National Museum of Natural History, MRC 166, Smithsonian Institution, Washington, DC 20013-7012 USA

**Keywords:** Phylogenetics, Plant evolution

## Abstract

*Typha* is a cosmopolitan aquatic plant genus that includes species with widespread distributions. It is a relatively ancient genus with an abundant fossil record dating back to the Paleogene. However, the details of its biogeographic history have remained unclear until now. In this study, we present a revised molecular phylogeny using sequences of seven chloroplast DNA markers from nine species sampled from various regions in order to infer the biogeographic history of the genus. Two clades were recovered with robust support. *Typha minima* and *T*. *elephantina* comprised one clade, and the other clade included the remaining seven species, which represented a polytomy of four robustly supported subclades. Two widespread species, *T*. *angustifolia* and *T*. *domingensis*, were revealed to be paraphyletic, indicating the need for taxonomic revision. Divergence time estimation suggested that *Typha* had a mid-Eocene crown origin, and its diversification occurred in the Middle and Late Miocene. Ancestral area reconstruction showed that *Typha* possibly originated from eastern Eurasia. Both dispersal via the Beringian Land Bridge and recent transoceanic dispersal may have influenced the intercontinental distribution of *Typha* species.

## Introduction

*Typha* L. (Typhaceae), also known as cattail, is a globally distributed aquatic plant genus. It grows in a variety of aquatic habitats on all continents except Antarctica^1^. Cattail is often dominant in wetlands and it is of concern in some regions due to its economic and ecological impact^2–4^. Several species are considered serious weeds that reduce biodiversity because they are highly productive by clonal growth, forming very large, persistent, and often monospecific stands^2,5,6^.

*Typha* includes 10–13 species, and most species have a widespread distribution^1,7^. Currently, taxonomic studies of *Typha* are mainly limited to a specific country or region, such as India^8^, Europe^9^, Iran and Pakistan^10,11^, Australia^12^, North America^13^, or China^14^. Due to the high morphological variability and frequent interspecific hybridization^1,15^, the taxonomy of *Typha* has been a longstanding debate. Traditionally, the genus was classified into two sections (*Ebracteolatae* and *Bracteolatae*) based on the presence or absence of bracteoles in the pistillate flowers, respectively^16,17^. In 1987, Smith^1^ made a taxonomic revision of *Typha* and recognized 8–13 species in six groups (without sections or subsections) based on the presence or absence of bracteole, in addition to morphological characteristics of the stigma and pollen grains. Fifteen new species were published after 1987^18^. All these species were local species, and none of them were presented with the support of molecular evidence. Some studies showed that the morphological characters of some new species overlapped with those of existing species. For example, Zhu^19^ measured a large number of specimens and found that the key characters of two new species, *T*. *tzvelevii* sp. nova and *T*. *joannis* sp. nova^20^, were remarkably similar to *T*. *laxmannii* and *T*. *orientalis*, respectively. Zhu therefore questioned the validity of the two new species^19^. Similarly, the validity of three endemic Chinese species (*T*. *przewalskii*, *T*. *davidiana*, and *T*. *changbaiensis*) was placed in doubt by two morphological studies^19,21^, which were supported by a molecular study with extensive sampling throughout China^22^. In a recently published handbook, Mabberley listed *Typha* with 10–12 species^7^. Therefore, we described *Typha* with 10–13 species based on the work of Smith^1^ and Mabberley^7^.

*Typha* is a relatively ancient genus. The earliest *Typha* fossil records that have been found were seeds assigned to *T*. *ochreaceae* Knobloch and Mai and *T*. *protogaea* Knobloch and Mai from the Late Cretaceous (Maastrichtian) period in Eisleben, Germany^23^. The earliest fossil record of *Sparganium* L. (the other genus of Typhaceae) is from the late Maastrichtian in Alberta, Canada^24^. In China, the earliest record of pollen grains assigned to Typhaceae was from the uppermost Maastrichtian (Senonian) to Paleocene sediments^25^. Both *Typha* and *Sparganium* have extensive and distinctive fossil records dating back to the Paleogene^26–29^. These fossil records can provide useful information for calibration in molecular dating to infer the biogeographical history of *Typha*.

A previous molecular phylogenetic study outlined the phylogenetic relationships among nine *Typha* species^30^. However, a recent study with broad sampling identified *T*. *angustifolia* as a paraphyletic species with two highly divergent lineages^31^. Therefore, it is necessary to reevaluate the molecular systematics of *Typha*. The intercontinental dispersal of several widespread *Typha* species has been recently investigated^31,32^, whereas the origin and diversification of the genus have remained unclear until now. It is time to reconstruct the historical biogeography of the *Typha* genus.

In this study, we used sequences from seven chloroplast DNA regions to reconstruct the phylogenetic tree of *Typha*. In addition, we estimated the evolutionary timescale of *Typha* based on fossil records in order to explore the historical biogeography of this cosmopolitan genus.

## Results

### Phylogenetic analyses

**Figure 1:**
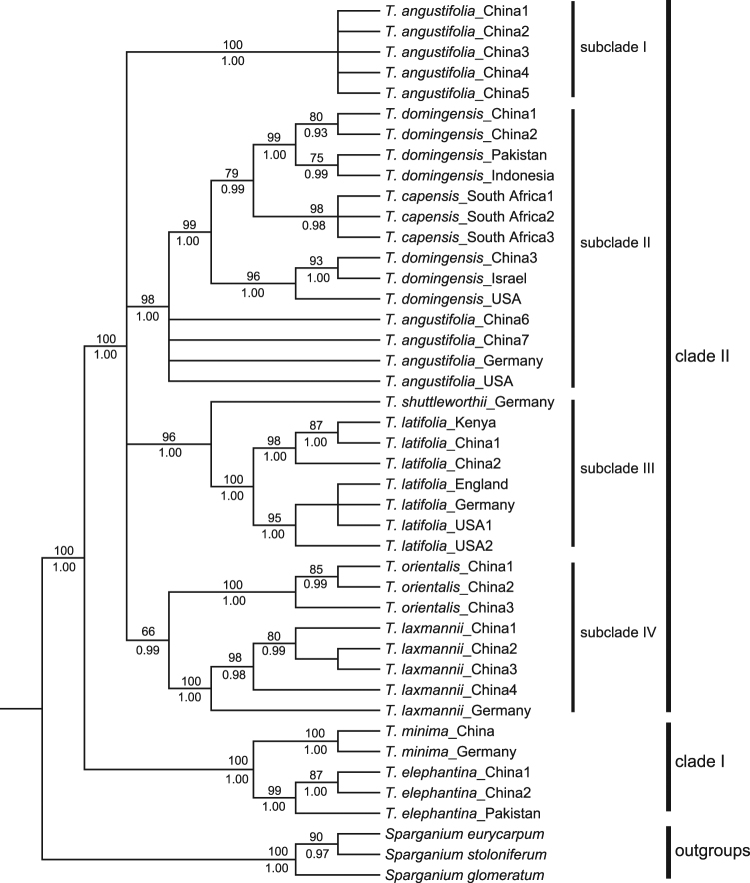
Bayesian consensus tree for *Typha* and three *Sparganium* species. The phylogenetic tree has been reconstructed based on seven chloroplast DNA regions (*atp*B-*rbc*L, *psb*A-*trn*H, *psb*D-*trn*T, *rpl*32-*trn*L, *rps*16 intron, *rps*16-*trn*K, and *trn*L-*trn*F). Numbers below the branches are Bayesian posterior probabilities (PP), and numbers above the branches are the ML bootstrap values (BS).

The aligned and concatenated sequences were 6,106 bp long with 988 variable sites. Of these, 426 were parsimony-informative. Phylogenetic relationships were inferred using maximum likelihood (ML) analysis and Bayesian inference (BI). The ML and BI trees were identical in topology (Fig. 1). The monophyly of *Typha* was strongly supported by both analyses (ML bootstrap support [BS] 100%, BI posterior probability [PP] 1.00). The genus was divided into two clades with strong support. The first clade (clade I) consisted of *T*. *minima* and *T*. *elephantina* (BS 100%, PP 1.00), and each species was determined to be monophyletic. The second clade (clade II; BS 100%, PP 1.00) included all remaining species and represented a polytomy of four robustly supported subclades. Subclade I (BS 100%, PP 1.00) included *T*. *angustifolia* only. Subclade II (BS 98%, PP 1.00) included *T*. *angustifolia*, *T*. *domingensis*, and *T*. *capensis*. Within this subclade, *T*. *domingensis* and *T*. *capensis* formed a highly supported group (BS 99%, PP 1.00), which was polytomic with three accessions of *T*. *angustifolia*, while *T*. *capensis* was nested in *T*. *domingensis*. Within subclade III (BS 96%, PP 1.00), *T*. *latifolia* was sister to *T*. *shuttleworthii* and it was further divided into two strongly supported groups. Subclade IV (BS 66%, PP 0.99) consisted of *T*. *orientalis* and *T*. *laxmannii*, which both formed their own monophyletic groups (Fig. 1).

### Divergence time estimation

**Figure 2:**
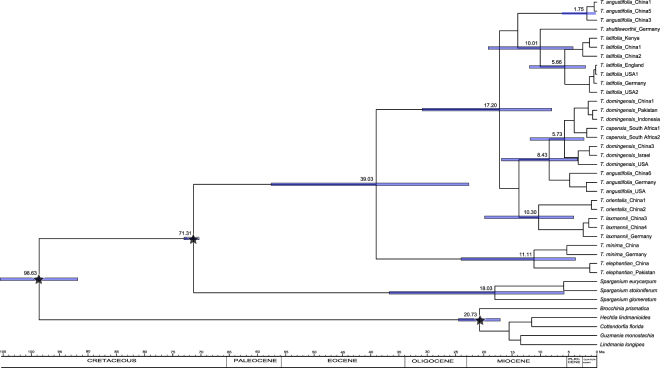
Chronogram of *Typha*, three *Sparganium* species, and five Bromeliaceae species inferred from BEAST. Blue bars represent the 95% highest posterior density intervals for node ages. Stars indicated three calibration points.

The respective crown ages of *Typha* and *Sparganium* were estimated to be 39.03 Mya (95% HPD: 22.64–57.60 Mya) and 18.03 Mya (5.79–36.69 Mya), respectively, based on combined data that included five Bromeliaceae sequences (Fig. 2). The beginning of diversification of the first clade, which included *T*. *minima* and *T*. *elephantina*, was dated to 11.11 Mya (3.78–24.01 Mya), and the second clade, which included the remaining species, was dated to 17.20 Mya (7.99–30.86 Mya). In the second clade, all three multiple-species subclades were estimated to begin to diversify in the Late Miocene (Fig. 2).

### Historical biogeography inference

**Figure 3:**
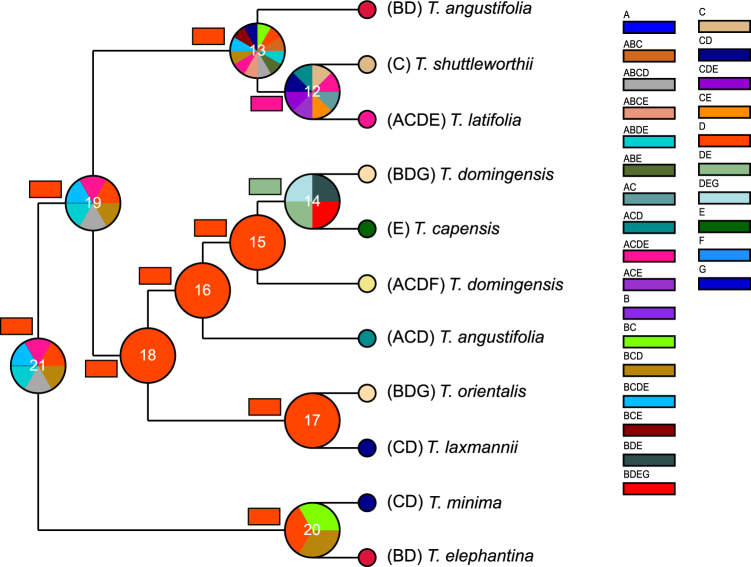
Reconstruction of the ancestral area of *Typha*. The pie charts at each node were obtained using S-DIVA analysis, and the rectangle charts beside each node were obtained from DEC analysis. The colors of the charts correspond to the most likely ancestral areas inferred. Letters represent the following biogeographic regions: (A) North America, (B) Indo-Pacific, (C) West Eurasia, (D) East Eurasia, (E) Africa, (F) South America, and (G) Australia.

Ancestral area reconstruction based on the dispersal-extinction-cladogenesis (DEC) analyses revealed East Eurasia as the ancestral area for the crown node of *Typha* genus, clade I, and clade II, while statistical dispersal-vicariance (S-DIVA) analyses determined that East Eurasia or other multiple areas were the ancestral area for the three nodes (Fig. 3, Supplementary Table S1). Both S-DIVA and DEC analyses supported East Eurasia as the ancestral area for the two multiple-species subclades, subclade II and IV, and multiple areas for subclade III, *T*. *latifolia*/*T*. *shuttleworthii* (Fig. 3). Eighteen dispersal events and only one vicariant event were revealed using the DEC analyses, while 22 dispersal events and two vicariant events were obtained using S-DIVA analyses.

## Discussion

In this study, we revealed that *Typha* is divided into two strongly supported clades. The first clade consists of two species, *T*. *minima* and *T*. *elephantina*. The second clade includes seven other species (Fig. 1). Our results are incongruent with a previous study that reported that *T*. *minima* is a clade and all other species form the other clade, including *T*. *elephantina*^30^. *Typha elephantina* and *T*. *minima* are morphologically distinct and can be easily distinguished from other species. *Typha elephantina* is distinct due to its robust habit, deep-set rhizome system, and stiff trigonal leaf blades^1,33^. *Typha minima* usually exhibits narrow, needle-like leaves and a stiff, unbranched central flower stalk^9^. Although their stems and leaves look very different from each other, *T*. *elephantina* and *T*. *minima* share four reproductive traits, including the presence of pistillate bracteoles, pollen in tetrads, filiform stigmas, and a gap between the male and female inflorescences, which have been used in taxonomic keys to identify *Typha* species^1,14,30^. Recently, Witztum and Wayne^34,35^ examined fiber cables in leaf blades of *Typha* species and found that the absence of fiber cables in leaf blades only occurs in *T*. *elephantina* and *T*. *minima*. The fact that *T*. *elephantina* and *T*. *minima* share the same five morphological characteristics suggests a closer affinity than has been previously considered. This supports our findings that *T*. *elephantina* and *T*. *minima* are sister species.

We found that *T*. *angustifolia* and *T*. *domingensis* are paraphyletic species, which suggests incongruence with a previous phylogenetic study of *Typha*^30^. Two highly divergent lineages were identified in *T*. *angustifolia*. One is subclade I, and the other nests in subclade II (Fig. 1). This was also observed by Ciotir and Freeland^31^, and it was named a core lineage and a divergent lineage by Freeland *et al*.^36^. Freeland *et al*.^36^ rejected the hypothesis that the divergent chloroplast DNA (cpDNA) lineage of *T*. *angustifolia* represents a cryptic species because it fell in the same genetic cluster as the core lineage based on nuclear genetic data from four microsatellite loci and the *LEAFY* gene. It was suggested that historical hybridization and introgression are the most likely explanation for this observation. In contrast, high divergence in nuclear *ADH* gene sequences was found in sympatric populations from both cpDNA lineages from northwest China^22^. This suggests that further investigation is needed to clarify the relationship between the two cpDNA lineages of *T*. *angustifolia*. In *T*. *domingensis*, two lineages were identified. One lineage was more closely related to *T*. *capensis* than to the other lineage, which formed a monophyletic group (Fig. 1). Similarly, Ciotir and Freeland^31^ observed paraphyly in *T*. *domingensis* and explained that it derived from incomplete lineage sorting following speciation. They also showed that these two lineages were distributed in different geographical ranges^31^. Further phylogeographic investigation is necessary in order to test whether or not *T*. *domingensis* includes cryptic species.

Although the earliest fossil of *Typha* is from the Late Cretaceous and fossils from the Paleogene are abundant^23,25–29^, these fossils do not exactly match extant *Typha* species. Therefore, we cannot rule out the possibility that these fossils are stem relatives, and we therefore treat them as representatives of the *Typha* stem lineage. The same treatment was used in some molecular dating studies^37–39^. Molecular dating results show that the crown origin of *Typha* occurred in the Middle Eocene (Fig. 2). East Eurasia was inferred as the ancestral area for the crown node of *Typha* in DEC analyses, and East Eurasia or other areas were inferred by S-DIVA analyses (Fig. 3). This suggests that crown-group *Typha* most likely originated in East Eurasia and then dispersed into other areas. It should be noted that this inference is not so convincing, because the relationship among the four subclades in clade II has not been fully resolved (Fig. 1).

Clade I consists of *T*. *minima* and *T*. *elephantina*, for which the most likely ancestral area was East Eurasia (Fig. 3), and the crown age was estimated to date to the Late Miocene (Fig. 2). In East Eurasia, the distribution of these two species is well-separated. *T*. *elephantina* is restricted to the south of the Qinghai-Tibetan Plateau (QTP), whereas *T*. *minima* is distributed north of the QTP. The QTP uplift is likely one of the factors that drove the divergence between these two species, although no consensus has been reached regarding the precise uplift phases of the QTP until now^40^. Clade II contained three intercontinentally dispersed subclades, including subclade II (i.e., *T*. *angustifolia*, *T*. *domingensis*, and *T*. *capensis*), subclade III (i.e., *T*. *latifolia* and *T*. *shuttleworthii*), and subclade IV (i.e., *T*. *orientalis* and *T*. *laxmannii*). Specifically, subclade II and III consisted of species that were intercontinentally dispersed between Eurasia and North America. The North Atlantic Land Bridge (NALB) and Beringian Land Bridge (BLB) served as migration routes for plants between Eurasia and North America^41^. The NALB facilitated taxa exchange until the Eocene, while the BLB contributed to intercontinental temperate taxa exchange until about 3.5 Mya^42–44^. The Late Miocene crown age of subclade II and III (Fig. 2) indicates that the BLB was a possible dispersal route for these temperate groups in *Typha*. In subclade II, the crown age of the *T*. *domingensis* lineage (including Eurasian and North American samples) was dated to about 3 Mya (Fig. 2), indicating a relatively recent transoceanic dispersal for *T*. *domingensis*. Similarly, transoceanic dispersal was also observed for *T*. *angustifolia* in a phylogeographical study based on sampling from Europe and North America^32^. In subclade III, *T*. *shuttleworthii* is restricted in Europe, and intercontinental dispersals occur in *T*. *latifolia*. High genetic divergence existed between the Asian and North American lineages^30^ (Fig. 1). Likewise, a phylogeographical study revealed that two recent *T*. *latifolia* colonizations have occurred: one from Asia into eastern Europe and the other from North America into western Europe^31^. The crown age of 5.66 Mya for *T*. *latifolia* (Fig. 2) suggested that the BLB was likely the route for *T*. *latifolia* dispersal between Asia and North America. For subclade IV, it was determined that *T*. *orientalis* dispersed from Asia to Australia based on having East Eurasia as the ancestral area. This dispersal route was also previously reported for other plants^45,46^. The time of dispersal into Australia varied widely amongst different taxa, even within single genera, such as *Cucumis*^47^. The time for *T*. *orientalis* dispersal is undetermined because no sample from Australia was included in this study.

## Methods

### Taxon sampling

A total of 43 samples were analyzed, including 40 from nine species of *Typha* and three outgroups from *Sparganium* species (Supplementary Table S2). Plant material was collected from Asia, North America, Europe, and Africa, and vouchers were deposited at the herbarium of Wuhan University (WH), South China Botanical Garden Herbarium (IBSC), and the United States National Herbarium (US). Detailed information regarding the samples and the associated GenBank accession numbers are listed in Supplementary Table S2.

### DNA extraction, amplification, and sequencing

Genomic DNA was extracted from silica-dried leaves using the DNA secure Plant Kit (Tiangen Biotech., Beijing, China) according to the manufacturer’s protocol. Seven cpDNA non-coding regions (*atp*B-*rbc*L, *psb*A-*trn*H, *psb*D-*trn*T, *rpl*32-*trn*L, *rps*16 intron, *rps*16-*trn*K, and *trn*L-*trn*F) were amplified and sequenced for this study. Sequences of the primers and their sources are listed in Supplementary Table S3. Polymerase chain reaction (PCR) was performed in a volume of 25 μL containing 10–30 ng genomic DNA, 0.1 μM of each primer, 0.2 mM of each dNTP, 2 mM MgCl_2_, and 0.6 U of ExTaq DNA polymerase (TaKaRa, Dalian, China). PCR reactions were performed in a Veriti 96-Well Thermal Cycler (Applied Biosystems, Foster City, USA) under the following conditions: 4 min at 95 °C, followed by 35 cycles of 45 s at 95 °C, 45 s at 55 °C, and 90 s at 72 °C, and then a final 10-min extension at 72 °C. The PCR products were purified and sequenced in both the 5′ and 3′ directions by the Beijing Genomic Institute in Wuhan, China.

### Phylogenetic analyses

All sequences were edited using Sequencher 4.1.4 (Gene Codes, Ann Arbor, MI, USA). Sequences were aligned using MAFFT 6.7^48^ and then manually checked using Se-Al (http://tree.bio.ed.ac.uk/software/seal/). Gaps were treated as missing data. The seven chloroplast DNA regions were concatenated for subsequent analyses. The phylogenetic relationships were inferred using ML analysis implemented in GARLI 2.0^49^. One-thousand bootstrap repetitions were performed to summarize the ML bootstrap support. BI implemented in MrBayes 3.1.2^50^ was also used for phylogenetic reconstruction. Two independent Markov chain Monte Carlo (MCMC) analysis runs were conducted simultaneously, and each run employed four chains. The analysis ran for 3 × 10^7^ generations with sampling at every 1,000 generations. Chain convergence was checked using Tracer 1.5^51^, and posterior probability values were generated from trees after excluding a burn-in of the first 25% of the trees. In the phylogenetic analyses, we assigned model parameters for each cpDNA region identified by Akaike information criterion (AIC) in jModeltest 2.1.7^52^. The K81uf + G model was selected for *psb*D-*trn*T, *rps*16-*trn*K, and *trn*L-*trn*F, while the HKY + I + G, F81 + I, K81uf + I, and TVM were suggested for *atp*B-*rbc*L, *psb*A-*trn*H, *rpl*32-*trn*L, and *rps*16 intron, respectively.

### Divergence time estimation

The divergence time between clades in *Typha* was estimated based on the concatenated sequence data containing seven cpDNA regions from 31 accessions of *Typha*, three species of *Sparganium*, and five species of Bromeliaceae. Sequences of the five Bromeliaceae samples in five cpDNA regions (*atp*B-*rbc*L, *psb*A-*trn*H, *rpl*32-*trn*L, *rps*16 intron, and *trn*L-*trn*F) were obtained from Givnish *et al*.^38^, and those in two cpDNA regions were treated as missing data. The divergence time estimate was conducted in BEAST 1.7.4^53^. The substitution model for each respective region was recalculated using jModeltest. The TIM + G model was selected for *atp*B-*rbc*L, the F81 + I model was selected for *psb*A-*trn*H, the TPM1uf + G model was selected for *psb*D-*trn*T and *rps*16-*trn*K, the GTR model was selected for *rps*16 intron, and the TVM + G model was selected for *rpl*32-*trn*L and *trn*L-*trn*F. Three calibration points were used. One was the stem age of *Typha*, which was a minimum age of 70 Mya based on fossil evidence. Although the earliest fossil of *Typha* dated to the Late Cretaceous and fossils from the Paleogene are abundant^23,25–29^, these fossils do not exactly match extant *Typha* species. Therefore, we cannot rule out the possibility that these fossils are stem relatives and treat them as representatives of the *Typha* stem lineage. The stem age of *Typha* has also been used in previous studies^37–39^. The detailed setting was: a lognormal prior with an offset of 70, a mean of 1.5, and a standard deviation of 0.5. The other two were the stem age of Typhaceae (100 ± 3.5 Mya) and the crown age of Bromeliaceae (19.1 ± 2.0 Mya), which were obtained from Givnish *et al*.^38^. MCMC analyses of 2 × 10^8^ generations were implemented, and every 1,000 generations were sampled. The first 25% of the generations were discarded as burn-in, and the parameters were checked using the program Tracer. Trees were summarized with Tree Annotator^53^.

### Reconstruction of ancestral areas

Ancestral area reconstruction was conducted using S-DIVA implemented in RASP 3.1^54^ and a likelihood model DEC implemented in Lagrange^55^. The analyses were conducted on a fully resolved topology from the BEAST analysis containing seven species, two lineages of *T*. *angustifolia*, and two lineages of *T*. *domingensis*. Seven geographical areas were defined based on the worldwide distribution of *Typha* according to Morse^56^: (A) North America, (B) Indo-Pacific, (C) West Eurasia, (D) East Eurasia, (E) Africa, (F) South America, and (G) Australia. The distribution of each species was determined based on our collecting localities and data from published papers^1,9,12,14,57,58^. Although *T*. *domingensis* from South America and Australia and *T*. *orientalis* from Australia were not included, these two geographical areas were also coded. For the DEC analysis, the dispersal probability between areas were set from 0.1 (for well-separated areas) to 1.0 (for adjacent areas) based on geological history and paleogeography reconstruction^59,60^ (Supplementary Table S4). The number of maximum areas was set to four because each lineage of *T*. *domingensis* and other species did not occur in more than four areas.

## Electronic supplementary material

Table S1-S4
