# Author Correction: Revised phylogeny and historical biogeography of the cosmopolitan aquatic plant genus *Typha* (Typhaceae)

**DOI:** 10.1038/s41598-020-67060-z

**Published:** 2020-06-12

**Authors:** Beibei Zhou, Tieyao Tu, Fanjiao Kong, Jun Wen, Xinwei Xu

**Affiliations:** 1 National Field Station of Freshwater Ecosystem of Liangzi Lake, College of Life Sciences, Wuhan University, Wuhan, 430072 P.R. China; 2 Key Laboratory of Plant Resources Conservation and Sustainable Utilization, South China Botanical Garden, Chinese Academy of Sciences, Guangzhou, 510650 P.R. China; 3 Department of Botany, National Museum of Natural History, MRC 166, Smithsonian Institution, Washington, DC 20013-7012 USA

Correction to: *Scientific Reports* 10.1038/s41598-018-27279-3, published online 11 June 2018

This Article contains errors.

In the Introduction section, the sentence,

“For example, Zhu^19^ measured a large number of specimens and found that the key characters of two new species, *T*. *tzvelevii* sp. nova and *T*. *joannis* sp. nova^20^, were remarkably similar to *T*. *laxmannii* and *T*. *orientalis*, respectively.”

should read:

“For example, Zhu^19^ measured a large number of specimens and found that the key characters of two new species, *T*. *tzvelevii* sp. nova and *T*. *joannis* sp. nova^20^, were remarkably similar to *T*. *orientalis* and, *T*. *laxmannii* respectively.”

In addition, Reference 20 was incorrectly given as:

Maveodiev, E. V. Two new species of *Typha* L. (Typhaceae JUSS.) from the Far East of Russia and from Mongolia. *Feddes Repertorium* **113**, 281–288 (2002).

The correct Reference is listed below:

Mavrodiev, E. V. Two new species of *Typha* L. (Typhaceae JUSS.) from the Far East of Russia and from Mongolia. *Feddes Repertorium* **113**, 281–288 (2002).

