# Supplementary material for: Revised phylogeny and historical biogeography of the cosmopolitan aquatic plant genus Typha (Typhaceae)
Source: Sci Rep. 2018 Jun 11;8:8813. doi: 10.1038/s41598-018-27279-3 (PMC5995854; doi:10.1038/s41598-018-27279-3)
Supplement: Supplementary file 1 — Table S1-S4 [file 41598_2018_27279_MOESM1_ESM.pdf]

Revised phylogeny and historical biogeography of the cosmopolitan aquatic plant genus *Typha* (Typhaceae)

Beibei Zhou[1,+], Tieyao Tu[2,+], Fanjiao Kong[1], Jun Wen[3,*], Xinwei Xu[1,*]

[1]National Field Station of Freshwater Ecosystem of Liangzi Lake, College of Life Sciences, Wuhan University, Wuhan 430072, PR China

[2]Key Laboratory of Plant Resources Conservation and Sustainable Utilization, South China Botanical Garden, Chinese Academy of Sciences, Guangzhou 510650, PR China

[3]Department of Botany, National Museum of Natural History, MRC 166, Smithsonian Institution, Washington, DC 20013-7012, USA

[+]these authors contributed equally to this work

[*]Correspondence and requests for materials should be addressed to X. X. xuxw@whu.edu.cn or J. W. wenj@si.edu

Table S1 Ancestral area reconstruction using the S-DIVA and DEC analysis based on the chronogram inferred using BEAST. Node number is indicated in Figure 3. PP is the posterior probability of the node. Letter codes represent biogeographic regions: A - North America, B - Indo-Pacific, C - West Eurasia, D - East Eurasia, E - Africa, F - South America, and G - Australia. Support for specific reconstructions are indicated by the integer to the right of the letter code. Reconstructions with less than 5% support were omitted.

Node	Age (Mya)		Ancestral areas				
	Mean	95% HPD	PP	S-DIVA		DEC	
				Area	Probability	Area	Probability
12	10.01	4.20-19.20	1.00	AC/CE/ACE/CDE/CD/ACD/C/ACDE	12.5/12.5/12.5/12.5/12.5/12.5/12.5/12.5	ACDE/C/CD/ACD/ CDE	27/22/18/9/9
13	--	--	0.90	ABDE/ABE/ABCD/ABCE/ACDE/BCD/BCDE/BCE/CD/BC/D/ABC	8.3/8.3/8.3/8.3/8.3/8.3/8.3/8.3/8.3/8.3/8.3	D/ACDE/CD/ABCD/BCDE/C/BCD/BC	29/17/13/7/7/7/6/5
14	3.99	1.38-8.53	1.00	BDEG/DE/DEG/BDE	25/25/25/25	DE/BDEG/BDE /DEG/D	28/18/16/16/8
15	5.73	2.27-11.80	1.00	D	100	D/DE/ACDF/BDEG/CD/AD	52/14/10/9/8/7
16	8.43	3.40-16.85	1.00	D	100	D/CD/AD	73/14/13
17	10.30	4.09-19.84	1.00	D	100	D	100
18	--	--	0.89	D	100	D	100
19	17.20	7.99-30.86	1.00	BCD/ABCD/ABDE/BCDE/ACDE/D	16.7/16.7/16.7/16.7/16.7/16.7	D/ACDE/ABCD/BCDE/CD/BCD	20/16/10/10/7/5
20	11.11	3.78-24.01	1.00	D/BCD/BC	33/33/33	D/BCD/CD/BD/ABCD/BCDE/BCDG/BCDF	28/17/12/12/8/8/8/8
21	39.03	22.64-57.59	1.00	D/BCD/ABCD/ABDE/BCDE/ACDE	16.7/16.7/16.7/16.7/16.7/16.7	D	8

Table S2 The collection information of samples used in this study and their GenBank accession numbers of seven chloroplast DNA regions.

Species	Collection site	Voucher No.	Code	atpB-rbcL	psbA-trnH	psbD-trnT	rpl32-trnL	rps16 intro	rps16-trnK	trnL-trnF
Typha angustifolia	China, Jinlin	Xu0068	*T. angustifolia*_China1	MG324303	MG430801	MG431016	MG430844	MG430887	MG430930	MG430973
Typha angustifolia	China, Qinghai	Tu519-1	*T. angustifolia*_China2	MG324304	MG430802	MG431017	MG430845	MG430888	MG430931	MG430974
Typha angustifolia	China, Shaanxi	Tu522	*T. angustifolia*_China3	MG324305	MG430803	MG431018	MG430846	MG430889	MG430932	MG430975
Typha angustifolia	China, Shaanxi	Tu523	*T. angustifolia*_China4	MG324306	MG430804	MG431019	MG430847	MG430890	MG430933	MG430976
Typha angustifolia	China, Hainan	Xu2012	*T. angustifolia*_China5	MG324307	MG430805	MG431020	MG430848	MG430891	MG430934	MG430977
Typha angustifolia	China, Xingjiang	Xu4155	*T. angustifolia*_China6	MG324318	MG430816	MG431031	MG430859	MG430902	MG430945	MG430988
Typha angustifolia	China, Xingjiang	Xu4450	*T.angustifolia*_China7	MG324319	MG430817	MG431032	MG430860	MG430903	MG430946	MG430989
Typha angustifolia	Germany, Bavaria	S. Renner s.n.	*T. angustifolia*_Germany	MG324320	MG430818	MG431033	MG430861	MG430904	MG430947	MG430990
Typha angustifolia	USA, Virginia	Wen9820	*T. angustifolia*_USA	MG324321	MG430819	MG431034	MG430862	MG430905	MG430948	MG430991
Typha domingensis	China, Yunnan	Xu2212	*T. domingensis*_China1	MG324308	MG430806	MG431021	MG430849	MG430892	MG430935	MG430978
Typha domingensis	China, Yunnan	Tu507	*T. domingensis*_China2	MG324309	MG430807	MG431022	MG430850	MG430893	MG430936	MG430979
Typha domingensis	China, Xinjiang	Xu2564	*T. domingensis*_China3	MG324315	MG430813	MG431028	MG430856	MG430899	MG430942	MG430985
Typha domingensis	Israel, Beersheba	S. Volis s.n.	*T. domingensis*_Israel	MG324316	MG430814	MG431029	MG430857	MG430900	MG430943	MG430986
Typha domingensis	Indonesia, West Java	Wen10698	*T. domingensis*_Indonesia	MG324311	MG430809	MG431024	MG430852	MG430895	MG430938	MG430981
Typha domingensis	Pakistan, Khyber Pakhtunkhwa	S.K.Marwat s.n.	*T. domingensis*_Pakistan	MG324310	MG430808	MG431023	MG430851	MG430894	MG430937	MG430980
Typha domingensis	USA, Texas	Moody1070	*T. domingensis*_USA	MG324317	MG430815	MG431030	MG430858	MG430901	MG430944	MG430987
Typha latifolia	China, Heilongjiang	Xu107	*T. latifolia*_China1	MG324324	MG430822	MG431037	MG430865	MG430908	MG430951	MG430994
Typha latifolia	China, Xingjiang	Xu4436	*T. latifolia*_China2	MG324325	MG430823	MG431038	MG430866	MG430909	MG430952	MG430995
Typha latifolia	USA, Virginia	Wen9821	*T. latifolia*_USA1	MG324328	MG430826	MG431041	MG430869	MG430912	MG430955	MG430998
Typha latifolia	USA, Alaska	Moody1077	*T. latifolia*_USA2	MG324329	MG430827	MG431042	MG430870	MG430913	MG430956	MG430999
Typha latifolia	Kenya, Kitale	X. Yan s.n.	*T. latifolia*_Kenya	MG324323	MG430821	MG431036	MG430864	MG430907	MG430950	MG430993
Typha latifolia	Germany, Bavaria	D/0053	*T. latifolia*_Germany	MG324327	MG430825	MG431040	MG430868	MG430911	MG430954	MG430997
Typha latifolia	UK, Bramhope	Reader s.n.	*T. latifolia*_England	MG324326	MG430824	MG431039	MG430867	MG430910	MG430953	MG430996

Typha orientalis	China, Zhejiang	Xu1065	*T. orientalis*_China1	MG324330	MG430828	MG431043	MG430871	MG430914	MG430957	MG431000
Typha orientalis	China, Hunan	Tu505	*T. orientalis*_China2	MG324331	MG430829	MG431044	MG430872	MG430915	MG430958	MG431001
Typha orientalis	China, Guangdong	Tu502	*T. orientalis*_China3	MG324332	MG430830	MG431045	MG430873	MG430916	MG430959	MG431002
Typha laxmannii	China, Guangdong	Tu526	*T. laxmannii*_China1	MG324333	MG430831	MG431046	MG430874	MG430917	MG430960	MG431003
Typha laxmannii	China, Shaanxi	Xu5239	*T. laxmannii*_China2	MG324334	MG430832	MG431047	MG430875	MG430918	MG430961	MG431004
Typha laxmannii	China, Jilin	Xu0069	*T. laxmannii*_China3	MG324335	MG430833	MG431048	MG430876	MG430919	MG430962	MG431005
Typha laxmannii	China, Qinghai	Tu518-1	*T. laxmannii*_China4	MG324336	MG430834	MG431049	MG430877	MG430920	MG430963	MG431006
Typha laxmannii	Germany, Bavaria	D/0222	*T. laxmannii*_Germany	MG324337	MG430835	MG431050	MG430878	MG430921	MG430964	MG431007
Typha minima	China, Shaanxi	Xu5235	*T. minima*_China	MG324338	MG430836	MG431051	MG430879	MG430922	MG430965	MG431008
Typha minima	Germany, Bavaria	D/0054	*T. minima*_Germany	MG324339	MG430837	MG431052	MG430880	MG430923	MG430966	MG431009
Typha elephantina	China, Yunnan	Tu506	*T. elephantina*_China1	MG324340	MG430838	MG431053	MG430881	MG430924	MG430967	MG431010
Typha elephantina	China, Yunnan	Tu515	*T. elephantina*_China2	MG324341	MG430839	MG431054	MG430882	MG430925	MG430968	MG431011
Typha elephantina	Pakistan, Khyber Pakhtunkhwa	S.K.Marwat s.n.	*T. elephantina*_Pakistan	MG324342	MG430840	MG431055	MG430883	MG430926	MG430969	MG431012
Typha capensis	South Africa, Hogsback	S. Compton s.n	*T. capensis*_South Africa1	MG324312	MG430810	MG431025	MG430853	MG430896	MG430939	MG430982
Typha capensis	South Africa, Free State	Wen10075	*T. capensis*_South Africa2	MG324313	MG430811	MG431026	MG430854	MG430897	MG430940	MG430983
Typha capensis	South Africa, KwaZulu-Natal	Wen10085	*T. capensis*_South Africa3	MG324314	MG430812	MG431027	MG430855	MG430898	MG430941	MG430984
Typha shuttleworthii	Germany, Bavaria	D/0055	*T. shuttleworthii*_Germany	MG324322	MG430820	MG431035	MG430863	MG430906	MG430949	MG430992
Sparganium stoloniferum	China, Heilongjiang	Xu3874	*S. stoloniferum*_China	MG324344	MG430842	MG431057	MG430885	MG430928	MG430971	MG431014
Sparganium eurycarpum	USA, Virginia	Strong s.n.	*S. eurycarpum*_USA	MG324343	MG430841	MG431056	MG430884	MG430927	MG430970	MG431013
Sparganium glomeratum	China, Heilongjiang	Xu3905	*S. glomeratum*_China	MG324345	MG430843	MG431058	MG430886	MG430929	MG430972	MG431015

Table S3 The primers of seven chloroplast DNA regions and their sources

Regions	Name and sequence (5'-3')	Reference
*atp*B-*rbc*L	*atpB*: GTG GAA ACC CCG GGA CGA GAA GTA GT	Hodges and Arnold, 1994
	rbcL: ACT TGC TTT AGT TTC TGT TTG TGG TGA	
*psb*A-*trn*H	trnH^{GUG}：CGC GCA TGG TGG ATT CAC AAT CC	Shaw et al., 2005
	psbA：GTT ATG CAT GAA CGT AAT GCT C	
*psb*D-*trn*T	psbD: CTC CGT ARC CAG TCA TCC ATA	Shaw et al., 2007
	trnT$^{(GGU)}$-R: CCC TTT TAA CTC AGT GGT AG	
*rpl*32-*trn*L	trnL$^{(UAG)}$: CTG CTT CCT AAG AGC AGC GT	Shaw et al., 2007
	rpL32-F: CAG TTC CAA AA A AAC GTA CTT C	
*rps*16 intron	rpS16F：AAA CGA TGT GGT ARA AAG CAA C	Shaw et al., 2005
	rpS16R：AAC ATC WAT TGC AAS GAT TCG ATA	
*rps*16-*trn*K	rpS16x2F2: AAA GTG GGT TTT TAT GAT CC	Shaw et al., 2007
	trnK$^{(UUU)}$x1: TTA AAA GCC GAG TAC TCT ACC	
*trn*L-*trn*F	e: GGT TCA AGT CCC TCT ATC CC	Taberlet et al., 1991
	f: ATT TGA ACT GGT GAC ACG AG	

Hodges SA, Arnold ML. 1994. Columbines: A geographically widespread species flock. Proceeding of the National Academy of Sciences of the United States of America 91: 5129-5132.

Shaw J, Lickey EB, Beck JT, Farmer SB, Liu W, Miller J, Siripun KC, Winder CT, Schilling EE, Small RL. 2005. The tortoise and the hare II: relative utility of 21 noncoding chloroplast DNA sequences for phylogenetic analysis. American Journal of Botany 92: 142-166.

Shaw J, Lickey EB, Schilling EE, Small RL. 2007. Comparison of whole chloroplast genome sequences to choose noncoding regions for phylogenetic studies in angiosperms: the tortoise and the hare III. American Journal of Botany 94:275-288.

Taberlet P, Gielly L, Pautou G, Bouvet J. 1991. Universal primers for amplification of three non-coding regions of chloroplast DNA. Plant Molecular Biology 17:1105-1109.

Table S4 Dispersal matrix among 7 areas used in the DEC analysis. Letters represent biogeographic regions: A - North America, B - Indo-Pacific, C - West Eurasia, D - East Eurasia, E - Africa, F - South America, and G - Australia. Dispersal probabilities among areas are given for 0 - 5 Ma, 5 - 33 Ma, 33 - 49 Ma, respectively.

	B	C	D	E	F	G
A	0.1/0.1/0.25	0.1/0.25/0.5	0.1/0.1/0.25	0.1/0.1/0.25	1.0/0.75/0.5	0.1/0.1/0.1
B	/	1.0/1.0/0.75	1.0/1.0/0.75	0.25/0.25/0.25	0.1/0.1/0.1	0.75/0.5/0.25
C		/	1.0/1.0/1.0	0.75/0.5/0.25	0.1/0.1/0.1	0.25/0.25/0.1
D			/	0.5/0.25/0.1	0.1/0.1/0.1	0.5/0.25/0.1
E				/	0.1/0.1/0.5	0.1/0.1/0.1
F					/	0.1/0.1/0.1
